# A panel of seven immune-related genes can serve as a good predictive biomarker for cervical squamous cell carcinoma

**DOI:** 10.3389/fgene.2022.1024508

**Published:** 2022-11-02

**Authors:** Junshang Dai, Yuwen Pan, Yili Chen, Shuzhong Yao

**Affiliations:** Department of Obstetrics and Gynecology, The First Affiliated Hospital, Sun Yat-sen University, Guangzhou, China

**Keywords:** immune-related genes, cervical cancer, prognosis, immune infiltration, tumor microenvironment

## Abstract

**Objective:** Cervical cancer is one of the most common gynecological malignancies. The interaction between tumor microenvironment and immune infiltration is closely related to the progression of cervical squamous cell carcinoma (CSCC) and patients’ prognosis. Herein, a panel of immune-related genes was established for more accurate prognostic prediction.

**Methods:** The transcriptome information of tumor and normal samples were obtained from TCGA-CSCC and GTEx. Differentially expressed genes (DEGs) were defined from it. Immune-related genes (IRGs) were retrieved from the ImmPort database. After removing the transcriptome data which not mentioned in GSE44001, IR-DEGs were preliminarily identified. Then, TCGA-CSCC samples were divided into training and testing set (3:1) randomly. Univariate Cox analysis, LASSO regression analysis and multivariate Cox analysis were used in turn to construct the signature to predict the overall survival (OS) and disease-free survival (DFS). External validation was performed in GSE44001, and initial clinical validation was performed by qRT-PCR. Function enrichment analysis, immune infiltration analysis and establishment of nomogram were conducted as well.

**Results:** A prognostic prediction signature consisting of seven IR-DEGs was established. High expression of NRP1, IGF2R, SERPINA3, TNF and low expression of ICOS, DES, HCK suggested that CSCC patients had shorter OS (P_OS_<0.001) and DFS (P_DFS_<0.001). AUC values of 1-, 3-, five- year OS were 0.800, 0.831 and 0.809. Analyses in other validation sets showed good consistency with the results in training set. The signature can serve as an independent prognostic factor for OS (HR = 1.166, *p* < 0.001). AUC values of 1-, 3-, five- year OS based on the nomogram were 0.769, 0.820 and 0.807. Functional enrichment analysis suggested that these IR-DEGs were associated with receptor interaction and immune cell activity. Immune infiltration analysis indicated that patients in high-risk group had lower immune infiltration, weaker immune function, and were more likely to benefit from immune checkpoint inhibitor therapy. Through qRT-PCR on clinical samples, expression of NRP1, IGF2R, SERPINA3 and TNF were significantly upregulated in tumor tissue, while ICOS and DES were significantly downregulated.

**Conclusion:** To conclude, the immune-related signature can provide strong support for exploration of immune infiltration, prediction of prognosis and response to immunotherapy through stratify CSCC patients into subgroups.

## Introduction

As the fourth most frequently occurring malignant tumor in females worldwide (Sung et al., 2021), cervical cancer is the leading cause of death and disability among various gynecological diseases, of which cervical squamous cell carcinoma is the main pathological subtype. Recently, although there have been big improvements in diagnostic techniques and treatment methods of cervical cancer, the clinical prognosis of cervical cancer, especially in patients with advanced stage, is still depressing. Unlike other malignant diseases such as lung or kidney cancer which happen on basically the elderly, highest incidence of cervical cancer was observed in young women. Undoubtedly, once the disease is recurrent or observed with metastasis, the overall survival is frustrating with only approximately 12 months (Banerjee, 2017). Therefore, on the one hand, we need to treat patients with surgery, chemotherapy and chemoradiotherapy; on the other hand, it is necessary to explore and identify a better risk stratification method which can accurately predict the prognosis of patients.

Tumor microenvironment (TME), including various immune cells like T cell, macrophages and B cells, is closely related to tumors’ malignant biological behaviors, such as distant organ migration and lymph node metastasis (Andersen et al., 2021; Bayik and Lathia, 2021; Li et al., 2021; Ozga et al., 2021; Shamseddine et al., 2021). For instance, three distinctive immunometabolism subtypes, immune suppressive-glycan metabolism subtype, immune inflamed-amino acid metabolism subtype and immune desert-endocrine subtype, were identified with implications for treatment methods and patients’ prognosis in ovarian cancer (Yang M. et al., 2021). Another research found that based on a 7-immune-related-gene signature, different immunocytes and their infiltration characterization was identified, as well as the immunotherapy response in hepatocellular carcinoma patients became predictable (Peng et al., 2021). Tumor-associated macrophages (TAMs) infiltration was found to be positively correlated with the stage of endometrial carcinoma. When co-culture TAMs overexpressing GAS5 with endometrial carcinoma cells, not only enhanced immune activities such as antigen presentation and cytotoxic T cells’ activation can be observed, but also attenuation of the immunosuppressive signal that originally exists in the TME at the same time (Tu et al., 2022). Although TME can serve as the front line for the body local antitumor immunity, and immune components in it do play a powerful anti-tumor effect most of the time, studies have shown that on the eve for distant metastasis of tumor cells, immune cells and their molecules have already played a pivotal role. For instance, the role of small extracellular vesicles in the process of tumor metastasis is one of the recent research hotspots. Some researchers have confirmed that, in head and neck squamous cell carcinoma, small extracellular vesicles carrying with CD73 can induce immune-suppression, which leads to immune escape by increasing the secretion of cytokines such as IL-6, IL-10, TNF-α and TGF-β (Lu et al., 2022). Different from treatment methods like surgery, radiotherapy or chemotherapy, the target of immunotherapy is the immune system. As for studies about immune checkpoints blockade, it has been reported that the B7-family immune checkpoint B7x can increase the expression of Foxp3, a Treg-specific transcription factor in CD4^+^ T cells, thereby reducing the efficacy of anti-CTLA-4 therapy through modulating the Akt/Foxo pathway (John et al., 2022).

Accumulated evidence has proved that the progression and prognosis of cervical cancer are related to a variety of factors. Recently, from the aspect of cell cycle and interaction with kinases, some researchers have found that, in the progression of cervical cancer, FBXO22 could promote G1/S phase progression and thus inhibit apoptosis of cancer cells through physically interacting with the cyclin-dependent kinase inhibitor p57Kip2 (Lin et al., 2022). As for TME and genomic instability can greatly affect patients’ prognosis by causing cancer, promoting tumor growth and increasing drug resistance, some hold the view that it is necessary to identify biomarkers which correlate with both TME and genomic instability to predict and evaluate patients’ survival and response to immunotherapy. Combined with comprehensive bioinformatics analysis, RIPOR2 was found to be closely related to tumor mutation burden (TMB), gene expression associated with DNA damage response (such as PARP1), TME-related scores, immune checkpoint activation, and immunotherapy efficacy (Xu F. et al., 2022). Lymph node metastasis of cervical cancer has always been a major obstacle in treatment. By proteomic sequencing of specimens from patients with positive and non-positive lymph node metastases, significantly increased expression of SPOP was found in patients with positive lymph node metastasis, and it can induce immune tolerance spatially and functionally through promoting PD-1 away from PD-L1 (Wu et al., 2022). Numerous prognostic signatures are emerging. The role of N6-adenosine methylation (m6A) in the TME has gradually attracted the attention of scholars. A prognostic signature based on m6A regulators (METTL16, YTHDF1, and ZC3H13) was identified to be an independent prognostic factor for cervical cancer, and AUC values of 3-, 5-, 10- year OS based on the signature were 0.666, 0.712 and 0.784 (Ji et al., 2022). Another study found that, a prognostic signature based on cuproptosis-related genes (ATP7A, DBT, DLAT, FDX1, GCSH, LIPT1, and PDHA1) had AUC values for 1-, 3-, five- year OS as 0.681, 0.698 and 0.677, respectively (Lei et al., 2022). For immune-related prognostic signature, previous studies have demonstrated that signature which based on the immune checkpoint molecule HLA-G driven DEGs (CD46, LGALS9, PGM1, SPRY4, CACNB3, PLIN2, MSMO1, and DAGLB) can conduct better risk prediction in 1-, 3-, 5-year OS in patients with cervical cancer (Xu H. H. et al., 2022). Besides, immune-related long non-coding RNA (lncRNA) was recommended for prognosis prediction as well. A six immune-related lncRNAs (AC006126.4, EGFR-AS1, RP4-647J21.1, LINC00925, EMX2OS, and BZRAP1-AS1) can perform good risk stratification of patients, while the immunotherapy response was completely negatively correlated with the risk scores.

However, their predictive power remains to be examined. As an open repository of discipline-level human immunology databases for translation and clinical research, Immunology Database and Analysis Portal (ImmPort) has been widely used for immune-related studies (Bhattacharya et al., 2018). Other database such as the Cancer Genome Atlas (TCGA), Gene Expression Omnibus (GEO), and Genotype-Tissue Expression (GTEx), which including transcriptome information, clinical data, etc., have been used on a large scale of countless studies and serve as a good solution to lack of sample size at the beginning of a study. Due to the lack of sufficient and quality-controlled sample studies, targets with both economical detection, specificity and sensitivity still cannot be identified, or narrowed to a more precise range. For this reason, we constructed a more clinically applicable model based on immune signature, consisting of multiple molecules, in order to provide better personalized treatment for patients with cervical cancer.

## Materials and methods

### Data acquisition

RNA-sequencing data and clinical profiles of 253 patients with CSCC and two normal tissue samples were downloaded from TCGA database (https://portal.gdc.cancer.gov/) on 24 June 2021. To expand the sample size, GTEx database (https://www.gtexportal.org/home/index.html) was used to obtain another 10 normal tissue samples’ RNA-sequencing data.

Then, gene expression was combined and normalized using the R package “limma”. 2,483 IRGs in total were related to human cancers, with the use of Immunology Database and Analysis Portal (ImmPort) database (https://www.immport.org/home) (Supplementary Table S1).

For external validation, gene array data and clinical information of 300 patients were downloaded from GSE44001, the platform of which was GPL14951, with the use of GEO database (https://www.ncbi.nlm.nih.gov/geo/). It was unnecessary to get ethics committee’s approval since the data above were downloaded from public databases.

### Identification of differentially expressed genes and immune-related genes

Using the R package “EdgeR”, IRGs which differentially expressed between the tumor samples and normal tissue samples were initially identified. With *p*-value<0.05 and |log2 (fold change) | > 1 as the cut-off values, 9,111 DEGs in total were discovered. Among these genes, 4,206 genes were down-regulated, and 4,905 genes were up-regulated. Then, the intersection in IRGs and DEGs was taken, and the obtained 607 genes were defined as IR-DEGs. After taking intersection with genes downloaded from GEO database, 584 IR-DEGs were used for subsequent analysis.

### Construction and verification of the immune-related signature with survival correlation

253 patients in total were randomly assigned to the training dataset (190 patients, 75% of all CSCC samples) and internal validation dataset (63 patients, 25% of all CSCC samples) sets, using the R package “caret”. Through univariate Cox regression analysis, 25 IR-DEGs which have a close correlation with patients’ OS (*p* < 0.05) were identified with the use of R package “survival” (*p* < 0.05). Subsequently, these IR-DEGs in the training dataset were adopted for the least absolute shrinkage and selection operator (LASSO). Using the R package “glmnet”, the most correlated genes were detected for predicting patients’ survival with penalty parameter adjustment by 10-fold cross validation. Following that, the candidate seven IR-DEGs with prognostic values were generated to construct the risk score signature with implementation of multivariate Cox regression analysis.

At the same time, the internal validation dataset was applied to validate whether the prognostic signature using these seven IR-DEGs have good reliability. Patients in these two datasets were stratified into the low- and high-risk groups according to the median risk score calculated by the constructed formula. With the use of Kaplan-Meier survival curves and log-rank test *via* the R packages “survival” and “survminer”, their survival was analyzed. Also, survival curves of each candidate genes in this signature were analyzed in to explore their predictive power respectively. The high-risk patients had shorter median survival than the low-risk patients (3.04 vs. 17.56 years, *p* < 0.001). As for the sensitivity and specificity of this prognostic signature in predicting 1-, 3-, and 5-year patients’ survival, receiver-operating characteristic (ROC) curves as well as the area under ROC curve (AUC) values was output utilizing the R package “survival ROC”. Then, the signature was further validated in the internal validation dataset (*n* = 63), the entire TCGA dataset (*n* = 253) and external GEO validation dataset (*n* = 300). Patients in each dataset were stratified into the low- and high-risk groups as described. Distribution of the survival status, risk score and candidate gene expression of patients in all datasets was analyzed as well.

To better illustrate the stability of this signature, information of DFS in the testing set, total TCGA-CSCC dataset and GSE44001 dataset were analyzed as well, through Kaplan-Meier survival curves, ROC curves and diagrams related to patients’ risk score.

### Biological feature exploration of prognosis-associated genes

To better understand the biological feature and function of the signature, Gene Ontology (GO) and Kyoto Encyclopedia of Genes and Genomes (KEGG) enrichment analysis were conducted using the R package “clusterProfiler”, “org.Hs.eg.db”, “enrichplot” and “ggplot2”. Adjusted *p*-value < 0.05 was selected as significantly enriched GO category or KEGG pathway.

### Screening of transcription factors and networks construction

With the help of cistrome online database, 318 transcription factors (TFs) in total were downloaded. The differentially expressed TFs correlated with DEGs were identified, using the R package “limma”, “dplyr”, “ggplot2” and “ggalluvial”. To construct the regulatory networks, Pearson correlation analysis was used as the main statistic methods. Among various filter criteria, false discovery rate (FDR) < 0.01 and correlation coefficient >0.4 were applied to detect nodes with high correlation. Then, the Search Tool for the Retrieval of Interacting Genes/Proteins (STRING) was used to visualize the functional protein association networks, and the minimum required interaction score was adjusted to high confidence, which may be known as 0.700, a proper balance between interaction correlation and gene identification.

### Correlation with clinicopathological features and development of nomogram

Determined by univariate Cox analysis, correlation between survival and clinical factors, including age, pGrade, pT, pN and risk score was analyzed. Then, multivariate Cox regression analysis was conducted to distinguish which factor has independent prognostic value. Meanwhile, Hazard ratios (HR) and corresponding 95% confidence interval (CI) were calculated in both analyses.

Next, nomogram was developed to better assess this immune-related signature’s predictive capability, which was a graphical method that intuitively combine various factors with risk score for comprehensive evaluation of patients’ survival. Using R packages “rms”, “survival” and “survcomp”, different clinicopathological features and their respect impact on patient survival were calculated and presented in the form of a graph. And according to C-index, ROC and calibration plots were analyzed to evaluate the nomogram’s predictive capability. Besides the predictive curve in calibration curve, the 45°line stood for the ideal prediction.

### Immune cells infiltration analysis based on risk grouping

With the use of the “gsva”, “GSEABase”, “ggpubr” and “reshape2” package, single sample gene set enrichment analysis (ssGSEA) was performed to calculate the scores of immune infiltrating cells and their functions in the whole TCGA-CSCC dataset. The “limma” package was used to analyze the difference of enrichment results between the two groups.

To explore immune cells infiltration in cancer foci, the Estimation of Stromal and Immune Cells in Malignant Tumours using Expression Data (ESTIMATE) algorithm was used to calculate the Stromal Score, Immune Score, ESTIMATE Score and Tumor Purity, combining transcriptome information of each patient. According to the specific infiltration situation of each tissue sample, the score represents the proportion of immune and stromal cells. The higher the score, the greater the proportion of the corresponding cell component. Both heatmap and vioplot were drawn to visually compare whether immune cell infiltration was different between low- and high-risk group. And expression levels of IRGs which contribute to the calculation for patients’ risk score were compared between two groups.

To further assess the infiltration of immune cells in tumor microenvironment, the Cell Type Identification by Estimating Relative Subsets of Known RNA Transcripts (CIBERSORT) were applied for analysis, with the use of R packages “preprocessCore”, “reshape2”, “ggpubr” and “limma”. As for CIBERSORT, based on transcriptome profiles from specimens composed of various components, 22 types of immune cells in total were included in lymphocytes infiltration analysis, such as B cells, T cells, nature killer cells (NKs) and macrophages.

### Identification of immune subtypes and prognostic value of the signature after immunotherapy

To clarify the relationship between immune cell infiltration in the microenvironment of tumor foci and patients’ risk scores calculated by the signature, the R package “ConsensusClusterPlus” was used for conducting clustering analysis. Among 22 types of immune cells in total, KM survival curves of each subtype as well as expression of seven IR-DEGs constituting the signature in different subtypes were studied.

With the use of the Tumor immune dysfunction and exclusion (TIDE) website (http://tide.dfci.harvard.edu), TIDE score and the tumor inflammation signature (TIS) score files of each patient with CSCC were downloaded and calculated. “limma” package was utilized to make comparisons among subgroups. Hereafter, time ROC curves were drawn by using R package “timeROC” to verify and compare accuracy of the signature we constructed and others.

### Bioinformatics verification of mRNA expression of genes in the signature

Utilizing GEO database, the R package “limma”, mRNA expression levels of each gene in the signature were analyzed. Genetic alteration profiles of risk genes in the signature were obtained from the cBioPortal database (https://www.cbioportal.org/).

### Collection of clinical specimens, RNA extraction and quantitative real-time polymerase chain reaction (qRT-PCR)

Our research followed the principles set forth in the Declaration of Helsinki, and the collection of tissues samples was approved by the Ethics Committee of the First Affiliated Hospital, Sun Yat-sen University (FAH-SYSU). Each patient was informed about the procedure and signed the relative informed consent. A total of 28 CSCC and 12 normal cervical tissue samples collected from January 2021 to January 2022 were obtained from patients who undergoing surgery at the FAH-SYSU. All these specimens used for experiments were validated by two pathologists, respectively. The inclusion criteria for CSCC samples were as follows: stage IA2 to IIA2, underwent at least radical hysterectomy, no preoperative chemotherapy, radiotherapy, or targeted therapy. The inclusion criteria for normal cervical samples were as follows: benign tissues confirmed by hematoxylin-eosin staining and immunohistochemistry, underwent radical hysterectomy, no other malignant diseases. Collection and handling of specimens in compliance with ethical guidelines.

Total RNA was extracted from the frozen specimens using Trizol as the manufacturer’s instructions (TAKARA, Japan). Each sample’s concentration and purity of the RNA extracts was measured by a NanoDrop-2000 spectrophotometer. Using the PrimeScript RT Master Mix following the manufacturer’s instructions (TAKARA, Japan), the reverse transcription into complementary DNA (cDNA) was accomplished. Amplification and quantification of cDNA was performed using a 7,500 Fast Real-Time PCR system (Applied Biosystems, United State) and SYBR Premix Ex Taq (TAKARA, Japan). cDNA samples were incubated under the setting as follows: 95°C for 2 min, followed by 39 cycles at 95°C for 20 s, 58°C for 30 s and 72°C for 30 s. The sequences of primers used in this study are listed in Supplementary Table S1. The whole procession of qRT-PCR was repeated at least three times. After normalization with reference to the house-keeping gene GAPDH expression, each gene’s relative expression was quantified by the 2−ΔΔCt method. The primer sequences were listed in Table 1.

**TABLE 1:** The information of primers used in qRT-PCR.

Gene	Sequences of primers
NRP1	FOR:ACGTGGAAGTCTTCGATGGAG
	REV:CACCATGTGTTTCGTAGTCAGA
ICOS	FOR:CAGGAGAAATCAATGGTTCTGCC
	REV:CCTTTTGTCTTAGTGAGATCGCA
DES	FOR:GAGACCATCGCGGCTAAGAAC
	REV:GTGTAGGACTGGATCTGGTGT
HCK	FOR:TCCGGGATAGCGAGACCAC
	REV:CCCGTCGTTCCCCTTCTTG
IGF2R	FOR:GTGACCAGCAAGGCACAAATC
	REV:CACCAAGTAGGCACCACTAAG
SERPINA3	FOR:CCTGAAGGCCCCTGATAAGAA
	REV:GCTGGACTGATTGAGGGTGC
TNF	FOR:GAGGCCAAGCCCTGGTATG
	REV:CGGGCCGATTGATCTCAGC
GADPH	FOR:GGAGCGAGATCCCTCCAAAAT
	REV: GGC​TGT​TGT​CAT​ACT​TCT​CAT​GG

### Statistical analysis

All statistical analyses were conducted using R language (version 4.0.5). The Wilcoxon test and one-way analysis of variance (ANOVA) were applied to compare two independent non-parametric samples or among multiple groups. Using the univariate and multivariate Cox analyses, independent prognostic factors closely related to prognosis were identified. Besides, the Kruskal–Wallis test was used to demonstrate the correlation between different risk group or clinicopathological features. As for survival analysis, the Kaplan-Meier survival curves and log-rank test were used to identify differences among groups. The Spearman method was used for calculating the correlation between IR-DEGs and immune cells. Results were regarded statistically significant when *p* < 0.05.

## Results

### Clinical characteristics of patients with cervical cancer

**FIGURE 1:**
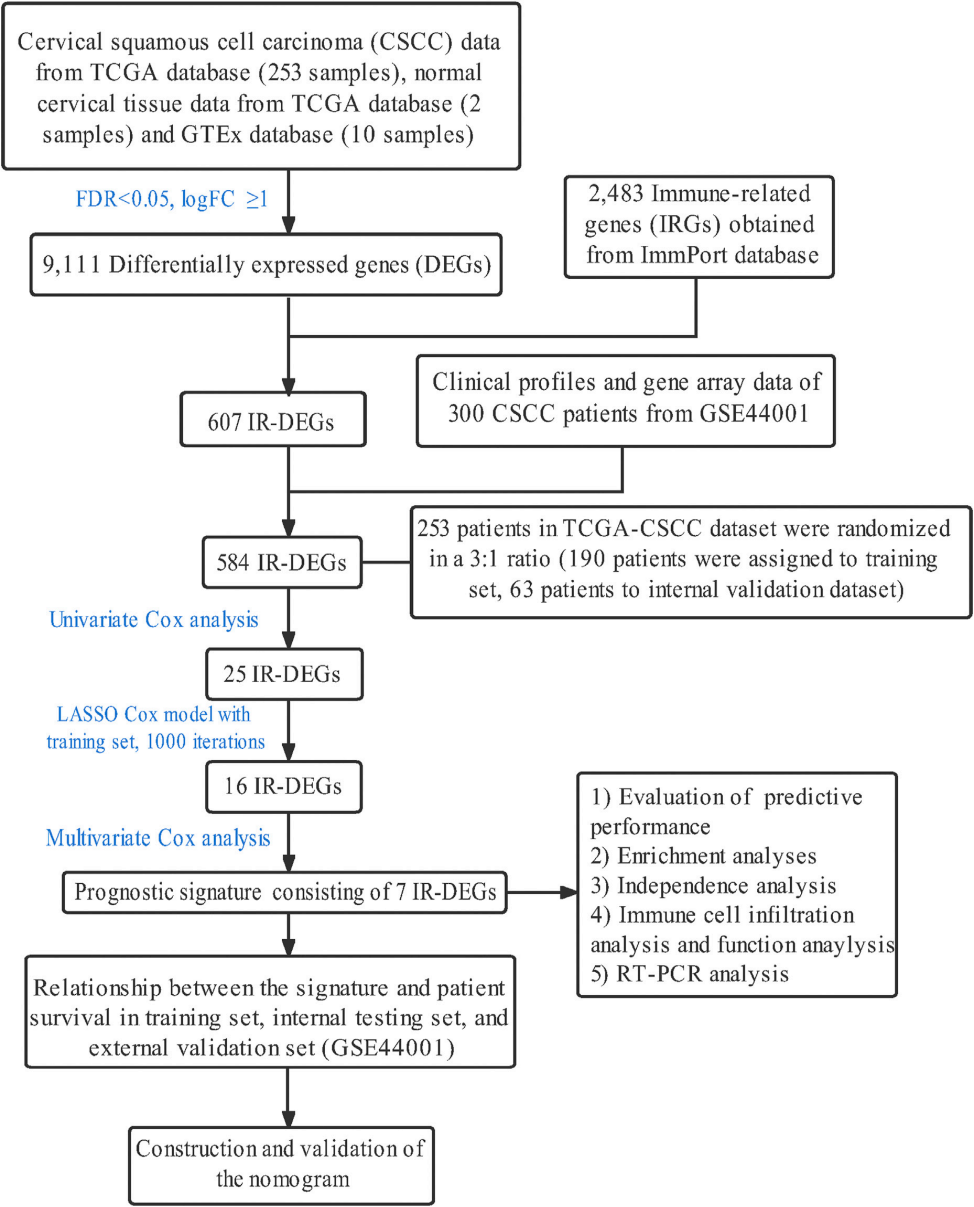
The flowchart of the present research.

The whole study was conducted as the flow chart (Figure 1). With the use of TCGA data portal, RNA-sequencing expression data and clinicopathological information of 253 CSCC patients were downloaded. Then, patients were randomly assigned to the training set (n = 190) and the testing set (n = 63). There were no significant differences (*p* > 0.05) in clinical variables between the patients in the training sets and testing sets (Table 2).

**TABLE 2:** Comparison between training set and testing set.

Characteristics	Training set (*n* = 190)	Testing set (*n* = 63)	*p* Value
Overall survival			
<3 years	131	46	
≥3 years	59	17	0.543
Survival status			
Alive	46	13	
Dead	144	50	0.563
Age			
<65 years old	164	54	
≥65 years old	26	9	0.905
Grade			
G1	8	3	
G2	87	23	
G3	73	30	
G4	1	0	
Unknown	21	7	0.589
T			
T1	85	26	
T2	40	18	
T3	13	5	
T4	8	1	
Unknown	44	13	0.723
M			
M0	72	30	
M1	5	2	
Unknown	113	31	0.158
N			
N0	80	25	
N1	35	15	
Unknown	75	23	0.967

### Identification of immune-related candidate prognostic genes

**FIGURE 2:**
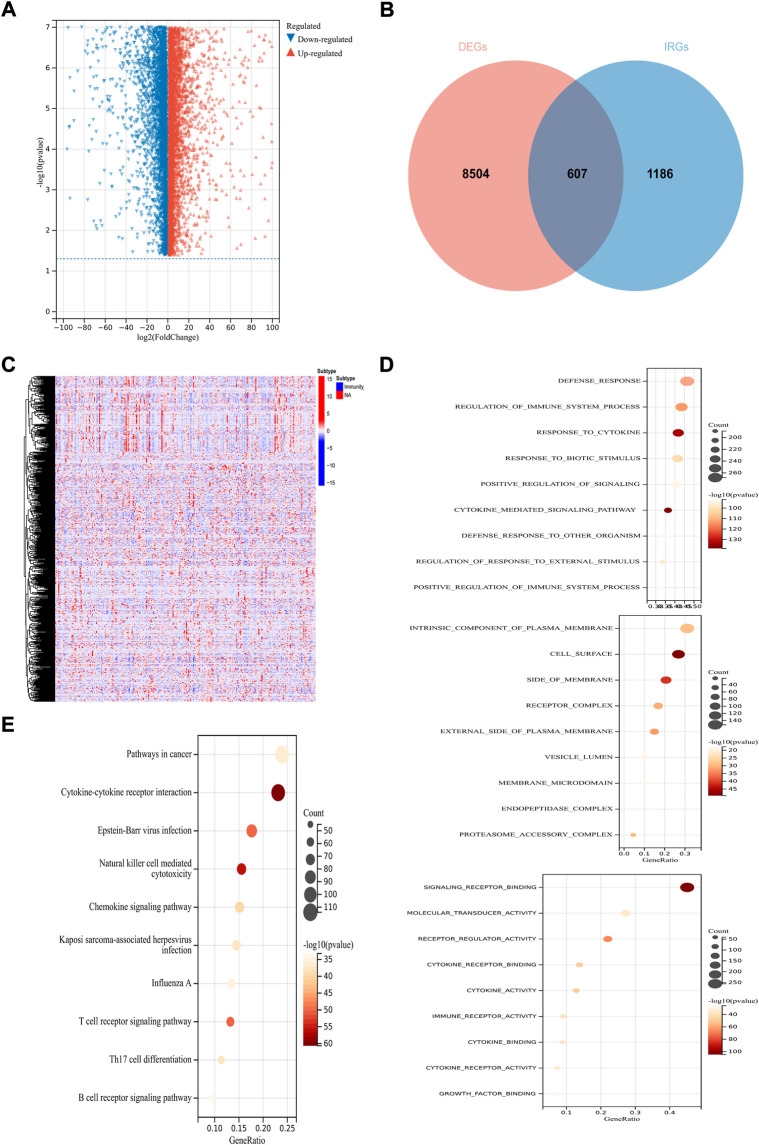
Identification of immune-related prognostic DEGs in CSCC. **(A)** The volcano plot of DEGs. **(B)** Venn diagram between DEGs and IRGs. **(C)**The heatmap of IR-DEGs. **(D)** GO enrichment analysis of IR-DEGs, from top to bottom are biological processes analysis, cellular components analysis and molecular functions analysis. **(E)** KEGG enrichment analysis of IR-DEGs.

Under the condition of adjusted *p*-value < 0.01 and |log2 (fold change) | > 1, 9,111 DEGs were obtained among 253 CSCC patients and two normal tissue samples from TCGA, and 10 standard cervix control samples GTEx. The volcano plot showed the expression levels of these genes (Figure 2A). Given the critical role of the immune microenvironment in cancer development, 607 IR-DEGs were identified after screened the ImmPort database (Figure 2B). After taking the intersection with the gene profiles in GSE44001, 584 IR-DEGs were kept for subsequent analysis. And the expression levels of these IR-DEGs were also visualized (Figure 2C). Then, GO enrichment analysis and KEGG enrichment analysis were conducted to preliminary explore the biological function of these IR-DEGs. GO analysis showed that the top three significantly enriched results in biological progresses were defense response, regulation of immune system process and response to cytokine; in cellular components were intrinsic component of plasma membrane, cell surface and side of membrane; in molecular function were signaling receptor binding, molecular transducer activity and receptor regulator activity (Figure 2D). The result of KEGG demonstrated that these IR-DEGs mainly enriched in pathways in cancer, cytokine-cytokine receptor interaction and Epstein-Barr virus infection (Figure 2E).

### Construction and validation of the immune-related prognostic signature

**FIGURE 3:**
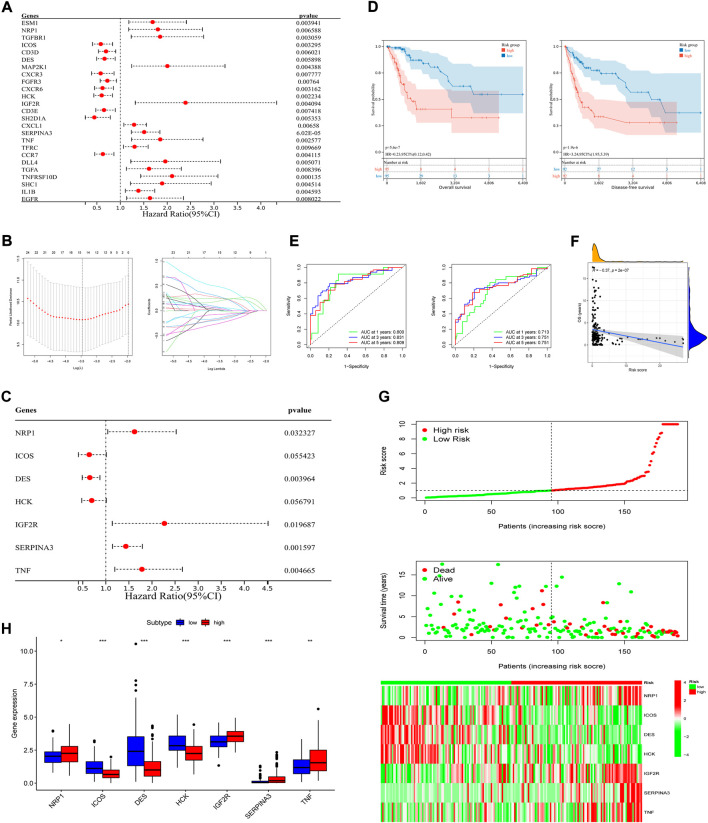
Construction of the prognostic signature and preliminary validation of its predictive power in training set. **(A)** Univariate Cox regression analysis of IR-DEGs in training set. **(B)** Select the optimal parameter (lambda) in the LASSO model. **(C)** Multivariate Cox regression analysis of IR-DEGs to identify seven IR-DEGs to construct a prognostic signature. **(D)** OS curves (left) and DFS curves (right) of high- and low-risk groups separated by the signature in training set. **(E)** Survival-dependent ROC curves of the training set corresponding to 1-, 3-, and 5-year OS (left) and DFS (right). **(F)** The correlation between OS and risk scores in training set. **(G)** The risk score distribution, survival status of patients, and expression heatmap of seven signature genes (from top to bottom). **(H)** Boxplot of expression levels of each gene of the signature in different risk groups in training set.

To explore the prognostic and therapeutic value of these 298 IR-DEGs, 25 prognosis-associated IR-DEGs were identified in training set with the use of univariate Cox analysis (Figure 3A). Then, in order to minimize the risk of overfitting, LASSO analysis was performed and 16 IR-DEGs were further screened out (Figure 3B). Within these 16 IR-DEGs, multivariate Cox analysis was used to construct the immune-related prognostic signature, which was a weighted linear combination of seven IR-DEGs according to their relative coefficient of expression level, and the risk score was calculated as follows:

Risk score = (0.48340 * NRP1) + (-0.44592 * ICOS) + (-0.42588 * DES) + (-0.36350 * HCK) + (0.81914 * IGF2R) + (0.36008 * SERPINA3) + (0.57810 * TNF).

According to the signature, four of the above IR-DEGs were known as high-risk genes (NRP1, IGF2R, SERPINA3, TNF, Coef >0), while other three IR-DEGs were associated with better prognosis (ICOS, DES, HCK, Coef <0) (Figure 3C). Next, these seven IR-DEGs expression data of each patient in the training set was used to calculate the risk score. Using the median risk score as the grouping standard, patients in the training set were divided into the high-risk group (n = 95) and low-risk group (*n* = 95). OS and DFS were compared between the high-risk group and low-risk group, and significant difference was found (P_OS_<0.001, P_DFS_<0.001, log-rank test), which indicated that patients in high-risk group had worse clinical prognosis than those in low-risk group (Figure 3D). To better illustrate the signature’s predictive function, AUC value of 1-, 3-, five- year OS were 0.800, 0.831 and 0.809, respectively. AUC value of 1-, 3-, five- year DFS were also calculated based on this signature, which were 0.713, 0.751 and 0.751, respectively (Figure 3E). In addition, the correlation with OS (Figure 3F), risk scores’ distribution, survival status, and expression of each gene based on the signature were analyzed, indicating its great predictive accuracy from another perspective (Figure 3G). Furthermore, in the total TCGA-CSCC dataset, expression of each gene of the signature was different in low- and high-risk group (Figure 3H).

To further clarify this prognostic signature’s applicability and accuracy, analysis was conducted in testing set, total TCGA-CSCC dataset and GSE44001 dataset, respectively. According to the risk scores calculated based on these seven IR-DEGs’ expression levels, patients in each dataset were classified into the high-risk group and low-risk group. As for patients in the testing set, 30 of them were in high-risk group and 33 of them were in low-risk group. Through Kaplan-Meier curves, when compared to patients in low-risk group, those in high-risk group had significant better prognosis in OS and DFS (P_OS_ = 0.039, P_DFS_ = 0.035, log-rank test) (Supplementary Figure S1A). The AUCs of OS were 0.699 at 1 year, 0.813 at 3 years, and 0.851 at 5 years. And the AUCs of DFS were 0.609 at 1 year, 0.762 at 3 years as well as 0.637 at 5 years (Supplementary Figure S1B). In the total TCGA-CSCC dataset, there were 125 patients were assigned to high-risk group and 128 patients were assigned to low-risk group. Not surprisingly, patients in high-risk group had shorter OS and DFS (P_OS_<0.001, P_DFS_<0.001, log-rank test) (Supplementary Figure S1C), with respective AUCs of 0.769, 0.820 and 0.807 for 1-, three- and 5-year OS, as well as 0.692, 0.741 and 0.738 for 1-, three- and 5-year DFS (Supplementary Figure S1D). The distribution of gene risk scores, survival status, gene expression levels, and correlation between OS and risk scores in both testing set and the total TCGA-CSCC dataset indicated that the signature, consisted of seven IF-DEGs, could make good assessment of patients’ prognosis (Supplementary Figure S1E–G).

In addition, predictive ability of the signature was applied to validate in GSE44001 for external validation. Using the above signature of seven IR-DEGs profiles, risk score of each patient were calculated and 128 of them were identified as high-risk patient, 172 of them were identified as low-risk patient. Similarly, great applicability in predicting DFS was indicated through Kaplan-Meier curves (*p* = 0.02, log-rank test) (Supplementary Figure S2A). Other perspectives such as the AUC of DFS (Supplementary Figure S2B), correlation between DFS and risk scores (Supplementary Figure S2C), gene risk scores’ distribution, survival status and levels of gene expression, great utility was shown (Supplementary Figure S2D).

### Application of the immune-related signature in classifying patients’ prognosis and clinicopathological features

**FIGURE 4:**
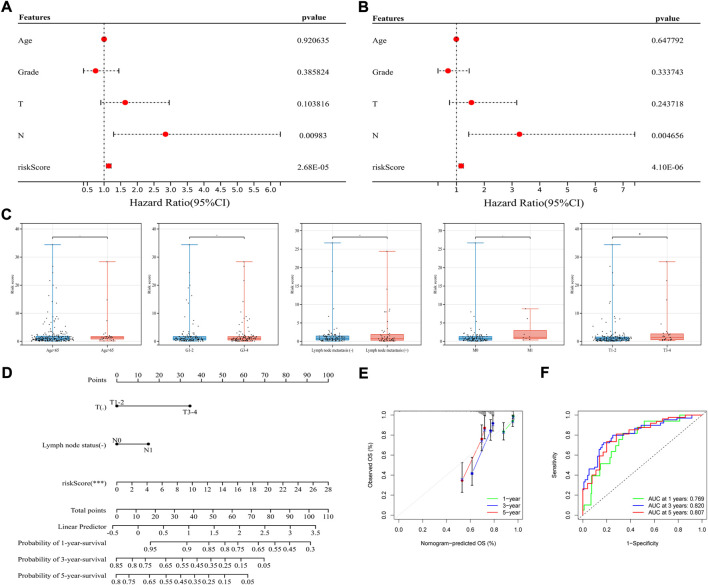
Application of the signature in classifying prognosis and clinicopathological features. **(A,B)** Univariate (left) and multivariate (right) Cox regression analysis in the whole TCGA-CSCC set. **(C)** Association between the signature and clinicopathological features, from left to right are age (the cut-off point is 65 years old), histological grade, lymph node metastasis, distant metastasis and T stage. **(D)** Construction of a nomogram integrated with clinicopathological characteristics. **(E)** The calibration curves based on the OS probability predicted by nomogram. **(F)** ROC curves of the whole TCGA-CSCC set corresponding to 1-, 3-, and 5-year OS based on the nomogram.

To figure out whether the signature can be used independently in predicting OS, using clinical profiles downloaded from the online database with detailed information of age, tumor grade, T stage, M stage and N stage, univariate and multivariate Cox regression analysis was conducted in total TCGA-CSCC dataset (Figure 4A). Then, multivariate Cox regression analysis was also conducted (Figure 4B). Combined these two analyses, a further explanation was that, besides N stage, the signature can serve as an independent prognostic factor for patients’ OS (risk score: HR = 1.166, 95%CI 1.092–1.245, *p* = 4.10e-06) (Supplementary Table S2). However, due to the limited information of known M stage (M0+M1, *n* = 110, <50%), the M stage data was excluded for either univariate Cox analysis or multivariate Cox analysis in the study.

To further examine whether this signature could classify patients with different clinical parameters, association between the signature and clinicopathological features was studied. Based on the signature, risk scores were calculated, and found to be significantly higher in patients with advanced T stage, indicating the signature was highly correlated with specific clinicopathological subtype. However, as for age, histological grade, lymph node metastasis, and distant metastasis, these subtypes could not be well divided by the signature (Figure 4C).

Based on the immune-related signature, a nomogram integrated with clinicopathological characteristics was constructed and analyzed. The nomogram showed a favorable discrimination (C-index = 0.820; HR = 2.718) and turned out that the greatest impact on this model came from risk score based on the signature. Frankly, patients with higher risk score have shorter survival, while with lymph node metastasis and larger tumor lesions have a tendency for worse prognosis (Figure 4D). According to the calibration curves, the OS probability predicted by nomogram had good consistency with the actual OS probability (Figure 4E). For example, the AUC value of 1-, 3-, and 5-year OS based on the nomogram was 0.769, 0.820, 0.807, and the AUC value of 1-, 3-, 5-year OS based on the signature was 0.800, 0.831, 0.809, indicating that the signature had stronger predictive power (Figure 4F). Earlier we have found that, most of the patients in TCGA-CSCC can be well divided by risk score. And as for characteristics such as tumor metastasis, only patients without distant metastasis can be performed more accurate prognostic prediction.

### Screening of transcription factors and functional annotation of the immune-related signature

**FIGURE 5:**
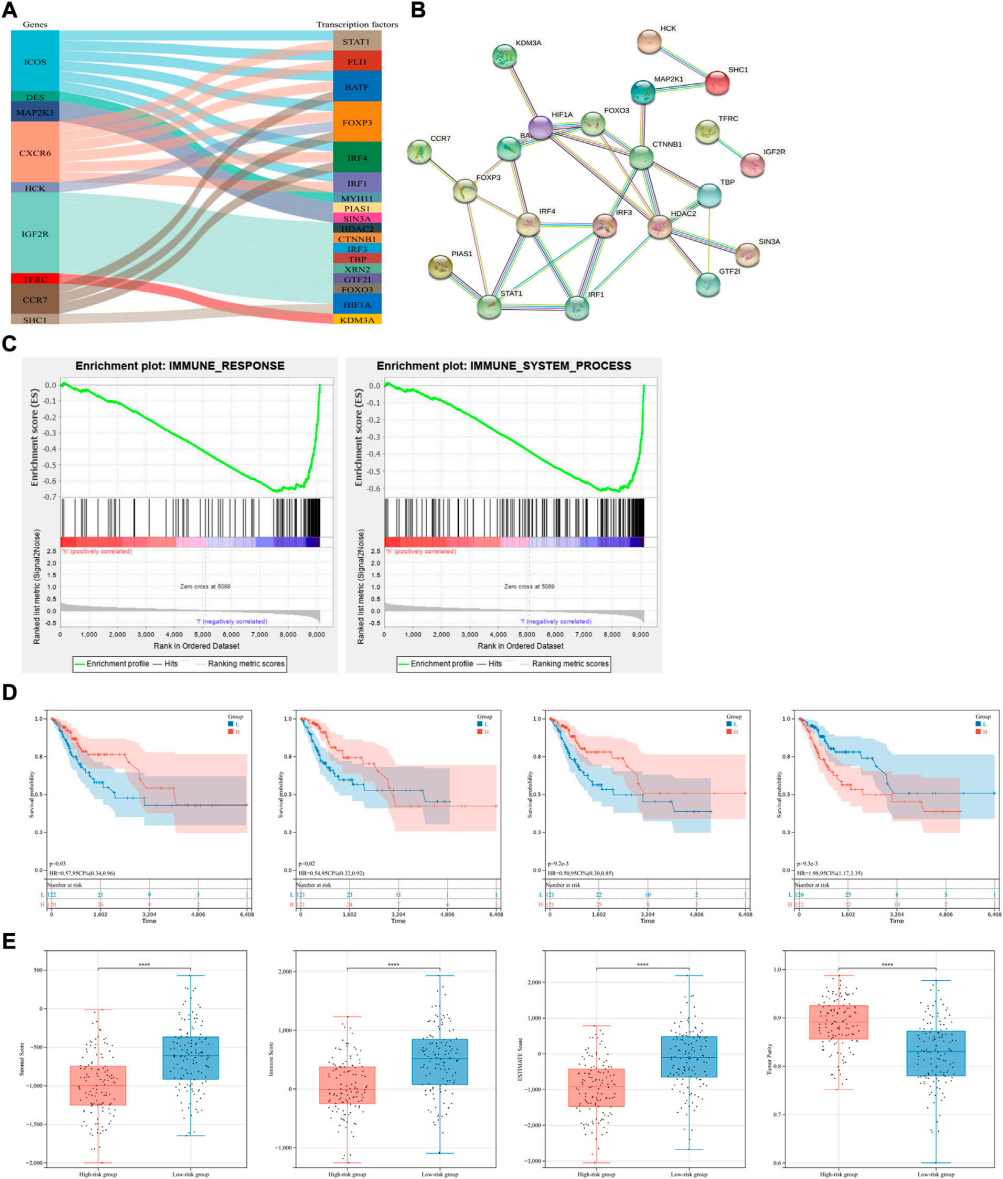
Transcription factors, functional annotation of the signature and exploration of tumor immune microenvironment. **(A,B)** TFs exploration and their regulatory relationship of seven candidate genes. **(C)** Gene Set Enrichment Analysis (GSEA) in the whole TCGA-CSCC set. Enriched immune response (left) and immune system process (right) pathways between the high- and low-risk groups were shown. **(D)** Conduction of survival analysis to assess the prognosis of patients with different scores of stromal cells, immune cells, ESTIMATE and tumor purity (from left to right). **(E)** The status of immune microenvironment in different risk group, stromal, immune, ESTIMATE scores and tumor purity were compared (from left to right).

The exploration of TFs in tumor cells can suggest the possible biological function of a gene to some extent. After comparing these 16 IR-DEGs and 318 TFs downloaded from the Cistrome database, expression of 18 TFs were identified as significant different between CSCC and normal tissues (Figure 5A). Correlation analysis was used to visualize the regulatory relationship with the use of Cytoscape software (Figure 5B).

The biological values of these 16 IR-DEGs in CSCC were further explored through enrichment analyses. For GO analysis, these IR-DEGs were significantly enriched in GO terms related to positive regulation of signaling, cell population proliferation, cell surface, anchoring junction, molecular transducer activity and signaling receptor binding (Supplementary Figure S3A–C). As for KEGG enrichment analysis, cytokine-cytokine receptor interaction, chemokine signaling pathway and MAPK signaling pathway were the top three enrichment pathways (Supplementary Figure S3D). Result of enrichment analyses showed that these IR-DEGs mainly associated with receptor interaction and immune cell activity.

Besides, Gene Set Enrichment Analysis (GSEA) was conducted in immune response gene set and immune system process gene set as well. And both these gene sets were enriched in low-risk group (Figure 5C).

### Exploration of tumor immune microenvironment and its correlation with the immune-related signature

With the use of ESTIMATE algorithm and based on the immune-related signature, stromal, immune, ESTIMATE scores and tumor purity were calculated in the whole TCGA-CSCC dataset on immune microenvironment (Supplementary Table S3). KM survival analysis showed that patients with higher stromal and ESTIMATE scores had better prognosis, and patients with higher tumor purity had shorter overall survival, indicating that the status of immune microenvironment can serve as an indicator of overall survival in CSCC patients (Figure 5D). To further explore the status of immune microenvironment in different risk group, stromal, immune, ESTIMATE scores and tumor purity were compared. The lower the risk score, the higher the stromal, immune and ESTIMATE scores. Opposite phenomenon was observed in the comparison of tumor purity (Figure 5E). Therefore, we can speculate that our prognostic model based on immune-related genes can predict the efficacy of immunotherapy to some extent.

### Immune cell infiltration analysis based on the immune-related signature

**FIGURE 6:**
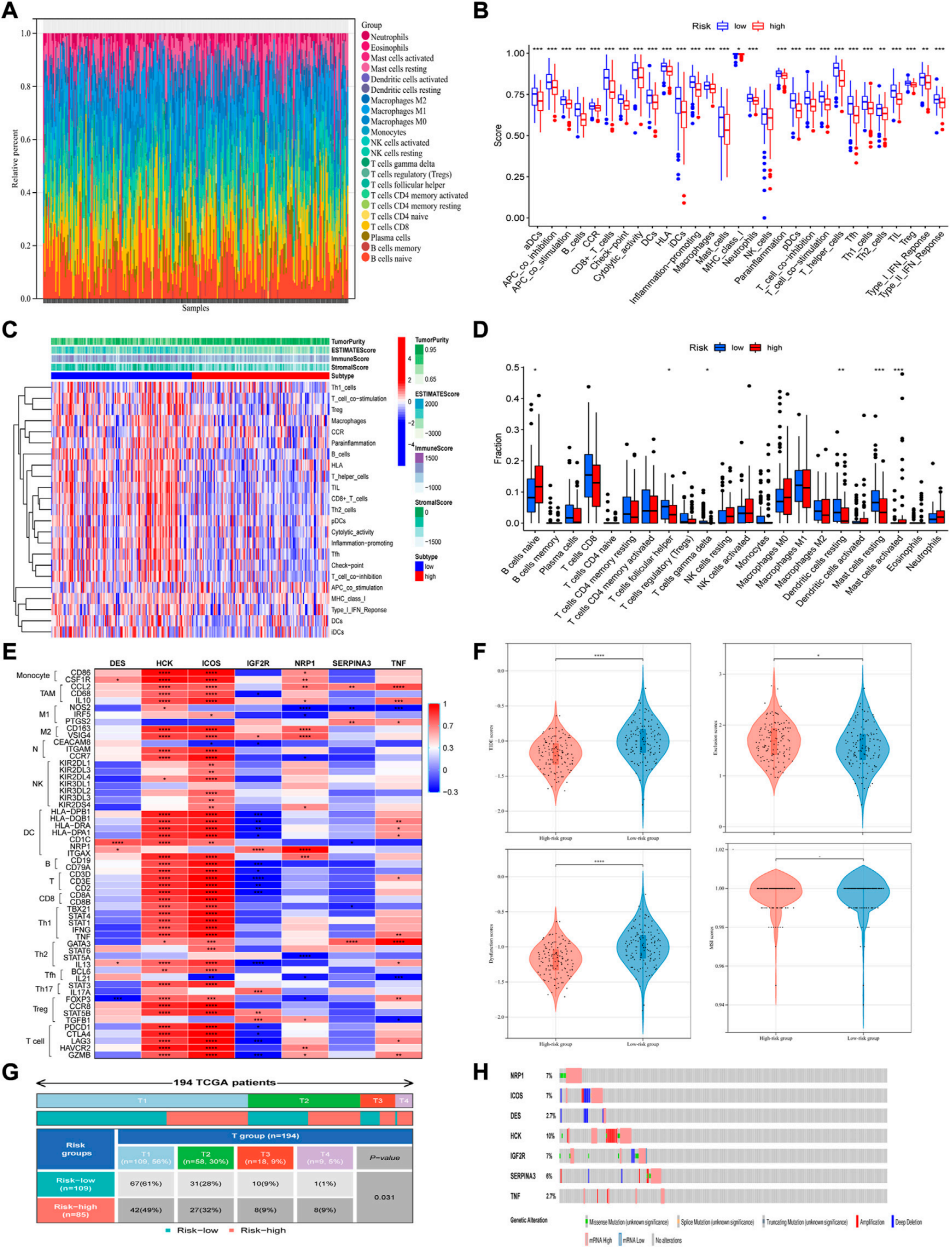
Immune cell infiltration analysis and related mutation analysis based on the signature. **(A)** The fraction of 22 immune cell types in total was analyzed with the use of CIBERSORT algorithm. **(B)** The immune functions were studied based on the ESTIMATE algorithm. **(C,D)** Both heatmap and boxplot showed the proportions of infiltrated immune components. **(E)** Expression of each candidate gene and its associated markers of immune cells. **(F)** The signature can perform better risk stratification of patients with different stages of tumor. **(G)** TIDE prediction was conducted in the whole TCGA-CSCC set, which analyzed from four aspects respectively: TIDE scores, Exclusion scores, dysfunction scores and MSI scores (from left to right, top to bottom). **(H)** Mutation analysis was performed using the cBioPortal online tool.

To further explore the tumor immune microenvironment, the CIBERSORT algorithm was utilized to give assessments on the infiltrated immune cell subtypes in all TCGA-CSCC samples. After screening the fraction of 22 immune cell types in total, it turned out that B cells naïve, T cell CD8, macrophages M0, macrophages M1 and mast cells resting were the top five most prevalent immune subtypes in the microenvironment (Figure 6A). Based on the result of ESTIMATE algorithm, related immune functions were studied in the whole TCGA-CSCC dataset. Besides NK cells, the scores of other immune functions and subpopulations were lower in the high-risk group (Figure 6B). Meanwhile, the proportions of infiltrated immune components were studied. It revealed that the proportions of T cells follicular helper, T cells gamma delta, dendritic cells resting and mast cells resting were lower in high-risk group. And the proportions of B cells naïve and mast cells activated were higher in high-risk group (Figures 6C,D). Taking the median expression of these infiltrated immune cells as the cutoff value, the lower the expression of macrophages M1, monocytes, T cells follicular helper, T cells CD4 memory activated, T cells CD8 and dendritic cells resting, the worse the overall survival. And the opposite phenomenon can be observed in NK cells activated and T cells CD4 memory resting (Supplementary Figure S3E). Besides, to better illustrate the interaction between these seven IR-DEGs and components of immune microenvironment, we explored the expression of each candidate gene and its associated markers of immune cells (Figure 6E). For example, DES positively correlated with dendritic cells (DCs)’ marker CD1C, IGF2R positively correlated with DCs marker NRP1, but negatively correlated with T cells’ marker CD2. NRP1 was associated with two types of macrophages; it positively correlated with M2 macrophages but negatively correlated with M1 macrophages and Th2 cells, proved by CD163, VSIG4, NOS2 and STAT6, respectively. Interestingly, SERPINA3 was positively correlated with TNF and TNF was positively correlated with CCL2, a marker of tumor associated macrophages. Other candidate genes such as HCK and ICOS had high correlation with most immune cells, suggesting that these two genes may play a double-edged role in immune infiltration. To further confirm whether this immune-related signature can stratify patients with certain characteristics, we performed subgroup analyses on their tumor size, grade of tumor, the status of distant metastasis and lymph node metastasis. Significantly difference between groups was observed only when we sub-grouped these patients by stages of tumor (Figure 6F). Taken these results together, it suggested that the immune-related signature may indicate the immune status of patients with CSCC.

### Effects of immunotherapy in different subgroups

To further explore the relationship between risk scores and patient outcomes, TIDE prediction was used to analyze each sample in the whole TCGA-CSCC dataset. According to the principle of the TIDE algorithm, the higher the score, the greater the potential of the patient to develop immune escape, and the less likely the benefit come from immunotherapy. We observed that, as for patients with high-risk scores, they had significantly lower scores of dysfunction and TIDE, but higher exclusion scores than patients with low risk scores (Figure 6G). These results indicated that high-risk patients with lower TIDE scores may benefit from immune-checkpoint inhibitor therapy.

### Mutation analysis, expression, and survival analysis of the candidate genes in the signature in patients with CSCC

Furthermore, to obtain more comprehensive genetic profiles of these seven candidate genes, cBioPortal online tool was used to perform mutation analysis. The highest mutation frequency among these candidate genes was reach up to 10% (HCK), and most of them have frequency between 6% and 7% (NRP1, ICOS, IGF2R and SERPINA3) (Figure 6H).

**FIGURE 7:**
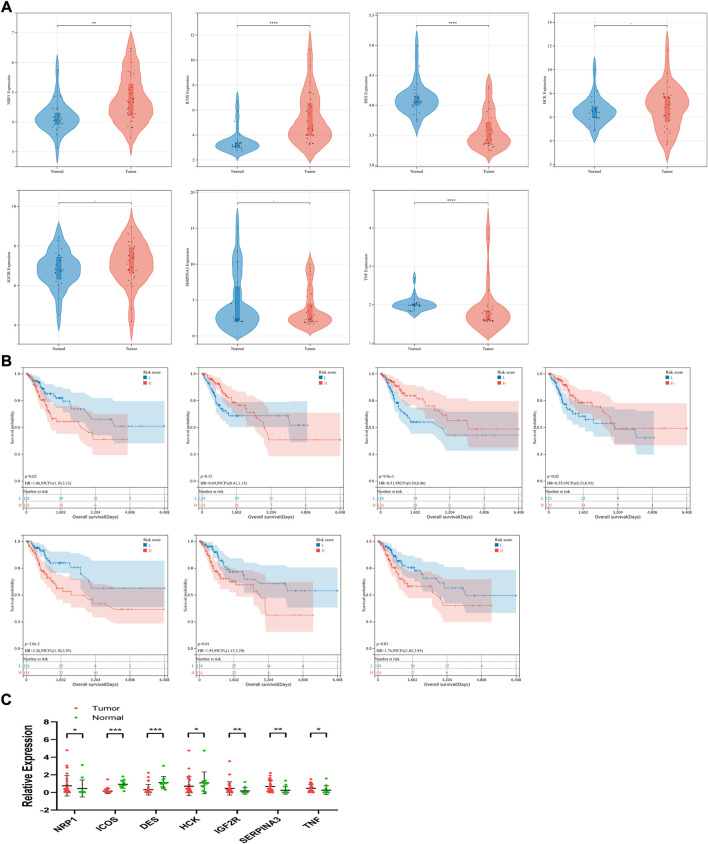
Expression levels, survival analysis and clinical sample validation of seven candidate genes. **(A)** Based on the signature, expression levels of each gene were compared in low- and high-risk group in GSE63514. **(B)** Survival analysis of each gene (from left to right, top to bottom: NRP1, ICOS, DES, HCK, IGF2R, SERPINA3 and TNF) was conducted in the whole TCGA-CSCC set. **(C)** Expression levels was analyzed using qRT-PCR.

To explore the expression of the seven candidate genes and their relationship with prognosis in patients with CSCC, survival analysis and expression verification were performed respectively. For patients with CSCC, analyses were performed in the GSE63514 dataset, which contained 24 cases of normal cervical tissue and 28 cases of *in situ* cervical cancer tissue. The expression levels of NRP1, ICOS, HCK, and IGF2R were significantly higher or tended to be higher in the high-risk group, while DES and SERPINA3 were lower in the high-risk group (Figure 7A). Meanwhile, in the whole TCGA-CSCC dataset, up-regulated expression levels of NRP1, IGF2R, SERPINA3 and TNF were correlated with worse clinical outcome, while high expression level of DES was considered to be a favorable factor associated with prognosis, but no relationship was observed between survival and ICOS and HCK (Figure 7B).

### Expression validation of candidate genes in the immune-related signature in clinical samples

The relevant clinicopathological parameters about the collected tumor sample were listed in Table 3. According to the qRT-PCR results, expression levels of NRP1, IGF2R, SERPINA3 and TNF were significantly upregulated in the comparison between *in situ* tumor samples and normal tissues in our clinical center. And expression of ICOS and DES were significantly downregulated in tumor patients (Figure 7C). From these results, validation of these candidate genes expression in our clinical samples was basically the same compared with our signature.

**TABLE 3:** The information of tumor samples for qRT-PCR.

Characteristics	Tumor samples (n = 28)	
Age		
<42 years old	7 (25%)	
≥42 years old	21 (75%)	
FIGO stage		
IA	5 (17.9%)	
IB	16 (57.1%)	
IIA	7 (25%)	
Depth of infiltration		
<1/2	7 (25%)	
≥1/2	14 (50%)	
Unknown	7 (25%)	
Degree of differentiation		
Low	6 (21.4%)	
Low to moderate	8 (28.6%)	
Moderate	6 (21.4%)	
Unknown	8 (28.6%)	

## Discussion

In this study, to construct an immune-related signature with better clinical applicability for patients with cervical cancer, the whole TCGA-CSCC dataset was randomly assigned in a 3:1 ratio. A cohort of 190 patients from the dataset was defined as the training set, and another cohort of 63 patients was defined as the internal validation set. Univariate analysis and LASSO analysis were performed in the training set in turn to construct a prognostic signature containing seven IR-DEGs, and the signature was validated in the internal validation set and the GSE44001 cohort (external validation set). Based on this immune-related signature, a nomogram was drawn with risk scores and other clinicopathological parameters, and it showed good accuracy in predicting the prognosis of patients with CSCC (AUC of 1-, 3-, 5-year OS = 0.769, 0.820, 0.807).

Functions and related mechanisms involved in several candidate genes have been described in previous studies. Neuropilin-1 (NRP1) was originally defined as a neuronal and endothelial receptor required for normal embryonic development and angiogenesis (Kawasaki et al., 1999). In recent years, its function as an immunomodulatory receptor has also been continuously explored (Acharya and Anderson, 2020). Studies have shown that NRP1 is upregulated in Tregs to regulate their stability and function of immune system in cancer patients (Liu et al., 2020). Battaglia et al. found that functions of Tregs expressing NRP1 appears varies with different anatomical location in CC patients (Battaglia et al., 2008). In addition to enhancing the functions of Tregs, NRP1 can also inhibit the immune memory generated by CD8^+^ T cells in the process of anti-tumor immunity, and anti-NRP1 treatment can improve the efficacy of anti-PD-1 immunotherapy (Leclerc et al., 2019). It also can inhibit tumor angiogenesis and thus upregulate anti-tumor immune responses (Hansen, 2013; Rizzolio et al., 2018; Teijeiro-Valiño et al., 2019). Furthermore, high expression of NRP1 is associated with poor prognosis in most cancer such as adrenocortical carcinoma, gastric adenocarcinoma and CC (Deng et al., 2021).

As a protein homologous to CD28 and CTLA-4, ICOS is defined as an inducible T cell co-stimulator, and usually upregulated in activated T cells (Hutloff et al., 1999), stimulating T cells’ proliferation and secretion of cytokines other than IL-2 (such as IL-4, IL-5, IL-10, TNA-α and IFN-γ, etc.) (Beier et al., 2000). Yang et al. found that low expression of ICOS is one of the risk factors for the prognosis of CSCC patients (Yang H. et al., 2021). In GOG-9929 study, CRT and sequential ipilimumab can increase memory T cell population. And the increased population of CD4^+^ T cells expressing ICOS and PD-1 was significantly associated with PFS and OS of patients (HR_PFS_ 0.806, P_PFS_ = 0.049; HR_OS_ 0.705, P_OS_ = 0.036) (Da Silva et al., 2020). Heeren et al. observed that in the context of CC lymph node metastases, CD8^+^ T cells were significantly increased, accompanied with elevated activation markers such as HLA-DR, ICOS, PD-1 and CTLA-4 (Heeren et al., 2015).

The relationship between HCK (hemopoietic cell kinase), DES (desmin), IGF2R (insulin-like growth factor 2 receptor), SERPINA3 (serpin peptidase inhibitor clade A member 3), TNF (tumor necrosis factor) and the prognosis of patient with CC has not been elucidated clearly. Previous studies have shown that HCK plays an important role in regulating the innate immune response, cell survival and proliferation as well as cell adhesion and migration (Kovács et al., 2014). And inflammatory mediators in microenvironment can also induce the activation of HCK in macrophages and neutrophils (Nelson et al., 2009; Poh et al., 2015). In acute myeloid leukemia, GSEA analysis suggests that HCK is enriched in immune- and inflammation-related pathways, and is associated with a variety of immune cell infiltration, including B cells, T cells, NK cells, TAMs, etc (Cheng et al., 2022). In pancreatic ductal adenocarcinoma, inhibition of HCK can target TAMs, thereby reducing the infiltration of immune cells, alleviating immunosuppression and improving chemotherapy efficacy (Creeden et al., 2020). DES, defined as the intermediate filament protein in striate muscle, is highly expressed in pancreatic cancer patients, and co-expression of ACTA2 and DES is positively correlated with lymph node metastasis (Li et al., 2019). IGF2R, represented as a type I transmembrane glycoprotein expressed ubiquitously in human tissues, is capable to inhibit tumor growth through binding to a wide range of ligands (Lobel et al., 1988; Oshima et al., 1988). Takeda et a l. found that depletion of IGF2R in CC interferes with the transport of M6P-labeled proteases from Golgi to lysosome, induces apoptosis by increasing misfolded proteins and promotes the production of active oxygen, and increase susceptibility to certain drugs such as cisplatin (Takeda et al., 2019). SERPINA3 is a protein of acute inflammatory response that positively correlates with HLA expression, and it also known as α1-antichymotrypsin (ACT) (Kloth et al., 2008). In prostate cancer, SERPINA3 promotes aerobic glycolysis and autophagy while inhibiting apoptosis of cancer cells (Xing et al., 2021). In glioblastoma and breast cancer, high expression of SERPINA3 is closely related to cancer cell proliferation, invasion, migration, and epithelial-mesenchymal transition, and thus leads to poor prognosis and tumor recurrence (Nimbalkar et al., 2021; Zhang et al., 2021). Similar phenomena can also be observed in endometrial cancer, liver cancer, colon cancer and so on (Cao et al., 2018; Ko et al., 2019; Zhou et al., 2019). TNF was defined as a multifunctional pro-inflammatory cytokine. On the one hand, it can exert anti-tumor effects by inducing apoptosis, participating in immune response, and inducing programmed cell necrosis. On the other hand, it promotes angiogenesis by inducing vascular endothelial growth factor, which also accelerates lymphatic tube formation, leading to lymphatic metastasis of tumor (Mocellin et al., 2005; Zidi et al., 2010). The specific mechanism of the above molecules in tumor-promoting in CC needs further study.

In our prognostic signature, survival analysis showed that patients in the low-risk group had better OS compared with the high-risk group. Risk stratification was performed respectively in patients diagnosed as T1-T2 or T3-T4 and found that our prediction signature could also predict their prognosis well. Recently, how to optimize the construction and predictive performance of prognostic signature has attracted the attention of scholars. (Iasonos et al., 2008; Chen C. et al., 2022; Pan et al., 2022; Yun et al., 2022). Not only based on the expression of selected genes but combined with other clinical parameters, the nomogram we constructed has better predictive performance than the signature alone (AUC_Signature_ = 0.80 vs AUC_Nomogram_ = 0.84), but its clinical applicability remains to be further evaluated.

In addition, ICI therapy has shown great clinical benefits since its introduction (Tang et al., 2018; Sinha et al., 2022). The application of PD-1/PD-L1 and CTLA-4 have been shown good curative effect on improving patients’ prognosis in hepatocellular carcinoma, non-small cell lung carcinoma, breast cancer and so on (Chae et al., 2018; Vaddepally et al., 2020; Lee et al., 2021; Chen Y. et al., 2022; Zhao et al., 2022). However, in terms of durable and effective response rate, a considerable number of patients fail to benefit from immune therapy, which accounts for 10%–40%. Some of them are primary drug resistance, while others relapse after a certain clinical remission, which is called acquired drug resistance (Zou et al., 2016; Sharma et al., 2017). Based on transcription profiles of patients, tumor immune evasion capacity is assessed by TIDE, which represents the degree of tumor immune dysfunction and exclusion, indicating the response for anti-PD-1/PD-L1 and anti CTLA-4 therapies (Fu et al., 2020). And it has been used in the prediction of immunotherapy effect in lung adenocarcinoma, colon adenocarcinoma, hepatocellular carcinoma and other cancer patients (Peng et al., 2021; Qiu et al., 2021; Zhang et al., 2022). Our study found that the TIDE scores of patients in high-risk group were significantly lower than low-risk group, suggesting that high-risk patients may be more likely to respond well to immunotherapy. The above results suggested that for CSCC patients, the signature we constructed can provide a good reference for developing individualized treatment plan or objectively assessing patient prognosis through performing good risk stratification based on the risk scores. It is widely recognized that the interaction between immune components and tumor cells can reshape TME (Liu et al., 2018). In our study, the immune enrichment scores of most immune cells in high-risk group were lower than those in low-risk group, that is, the immune activity of high-risk patients was inhibited, thereby facilitating the proliferation of tumor cells and making worse prognosis. Based on the CIBERSORT algorithm, we found that our signature was significantly correlated with the infiltration of DCs and mast cells. Combined with the scores of immune functions in both risk groups, we speculated that the signature may play an important role in regulating the immune TME, which may be mainly achieved by affecting the functions of DCs and mast cells. Specific to each gene, IGF2R was positively correlated with the infiltration level of DCs, as well as NRP1 was positively correlated with the infiltration level of M2 macrophages and negatively correlated with M1 macrophages. Such findings were consistent with previous studies (Petrillo et al., 2015). One study showed that the maturation of DCs can induce the production on CD4 +, CD8 + T cells and activated NKs, making it to become candidate target for immunotherapy of cervical cancer (Dhandapani et al., 2021). Some scholars also found that CXC chemokines in the TME play an important role in regulating the transportation of immune cells and activities of tumor cell. The expression of CXCL9/10/11 and CXCR3 in CC patients was significantly correlated with immune cells such as CD8^+^ T cells, CD4^+^ T cells, neutrophils and DCs (Kong et al., 2021). For M2 macrophages, it was found that NFATc1 mediates the secretion of IL-10 by regulating the c-myc/PKM2 pathway, and thus inducing TAMs’ polarization toward M2 macrophages, promoting the proliferation, migration and invasion of CC cells (Tan et al., 2022). In contrast, exosome-mediated miR-423–3p can inhibit the M2 polarization of TAMs through CDK4-mediated STAT3 phosphorylation, thereby inhibiting the proliferation and migration of CC cells (Yan et al., 2022). In summary, high-risk patients have a TME characterized by higher infiltration of DCs and M2 macrophages, which promotes the development of CC and makes them more likely to benefit from immunotherapy.

Although the immune-relate signature composed of seven IR-DEGs can perform better stratified analysis on the prognosis of CC patients, there are some limitations should be aware of. First of all, the sample size of the external validation set we used to conduct qRT-PCR was not large enough. Second, the current evaluation of this signature’s prediction performance still based on the online databases. However, the occurrence and development of CC is a multi-factor, continuous and gradual process. More prospective studies should be conducted as well as the sample size should be expanded in the future to perform a more comprehensive analysis. Third, the nomogram we developed contains less information, which may cause a deviation compared with the actual situation. Finally, the varying infiltration of immune components in CC patients suggests that the differences in immune-related signatures can serve as an intrinsic feature, which can be utilized to reflect changes individually with important clinical implications.

## Conclusion

Our study developed an immune-related signature which had a good performance in predicting prognosis for CSCC patients. After preliminary verification of the predictive performance of the signature, we conducted an exploration of the immune microenvironment of patients in low- and high-risk groups through immune infiltration analysis, TIDE analysis, TMB analysis and so on. Based the signature, we developed a nomogram with other clinical parameters and found it may provide better predictive power than the signature alone. We observed that patients in low-risk group had higher scores of immune functions and subpopulations. The correlation between each candidate gene and the expression of immune cells markers was also explored. Combine our findings, this study provides an accurate method for evaluating the prognosis of CSCC patients, discovering immune microenvironment signature of patients in different risk group, and exploring the potential value of immunotherapy.

## Data Availability

The original contributions presented in the study are included in the article/Supplementary Material, further inquiries can be directed to the corresponding author.

## Abbreviations

CSCC, Cervical squamous cell carcinoma; TCGA, The Cancer Genome Atlas; GTEx, Genotype-Tissue Expression; GEO, Gene Expression Omnibus; ImmPort, The Immunology Database and Analysis Portal; DEGs, Differentially expressed genes; IRGs, Immune-related genes; LASSO, Least absolute shrinkage and selection operator; qRT-PCR, Quantitative real-time polymerase chain reaction; TME, Tumor microenvironment; TAMs, Tumor-associated macrophages; TMB, Tumor mutation burden; m6A, N6-methyladenosine; lncRNA, Long non-coding RNA; GO, Gene Ontology; KEGG, Kyoto Encyclopedia of Genes and Genomes; TFs, Transcription factors; FDR, False discovery rate; ssGSEA, Single Sample Gene Set Enrichment Analysis; ESTIMATE, Estimation of Stromal and Immune Cells in Malignant Tumours using Expression Data; CIBERSORT, Cell Type Identification by Estimating Relative Subsets of Known RNA Transcripts; TIDE, Tumor immune dysfunction and exclusion; TIS, Tumor inflammation signature; FAH-SYSU, The First Affiliated Hospital, Sun Yat-sen University; NRP1, Neuropilin-1; ICOS, Inducible T cell co-stimulator; DES, Desmin; HCK, Hemopoietic cell kinase; IGF2R, Insulin-like growth factor 2 receptor; SERPINA3, Serpin peptidase inhibitor clade A member three; TNF, Tumor necrosis factor.
