# Supplementary figures and images for: A panel of seven immune-related genes can serve as a good predictive biomarker for cervical squamous cell carcinoma
Source: Front Genet. 2022 Nov 2;13:1024508. doi: 10.3389/fgene.2022.1024508 (PMC9667556; doi:10.3389/fgene.2022.1024508)

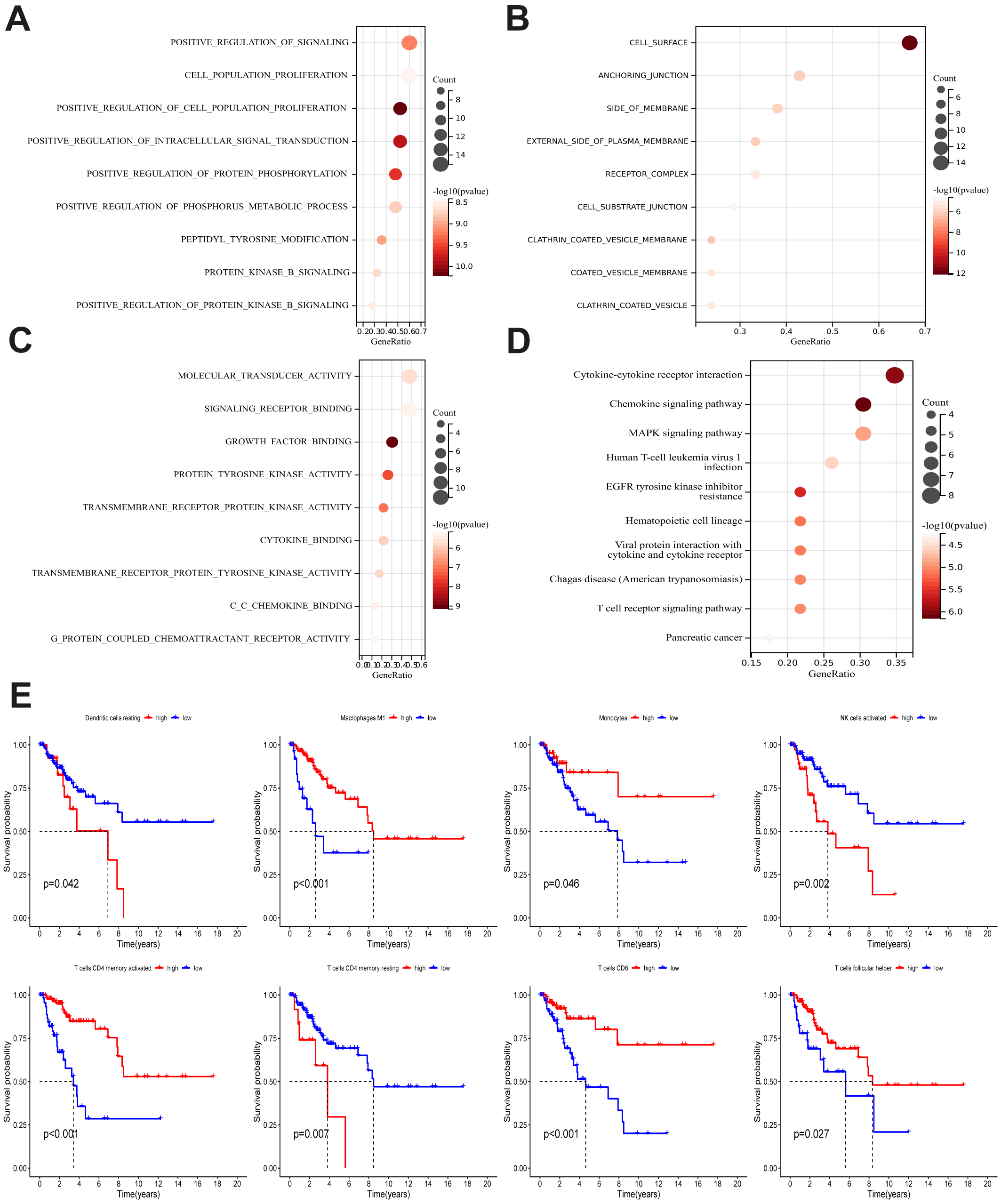

Supplement: Supplementary file 2 [file Image3.tif]

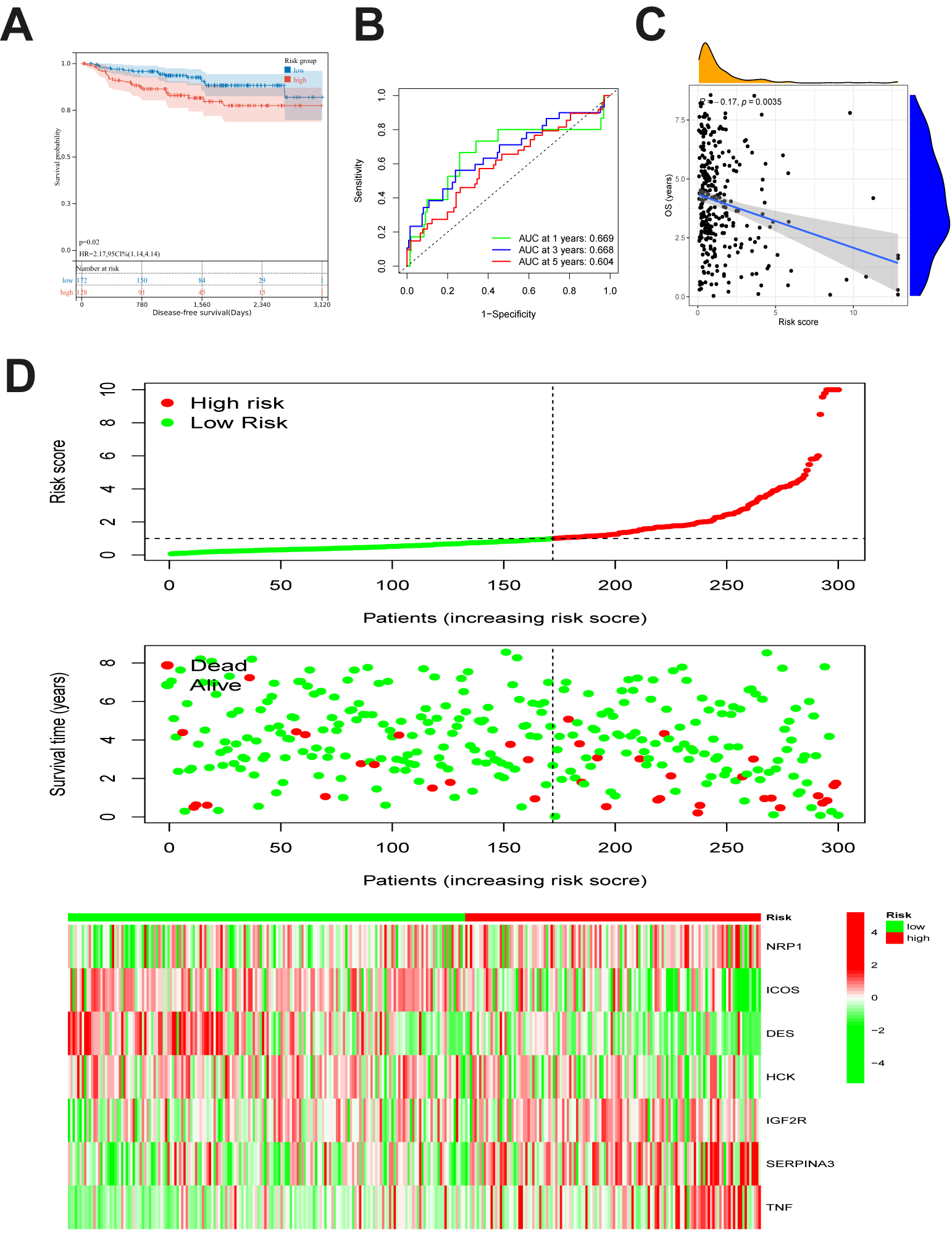

Supplement: Supplementary file 3 [file Image2.tif]

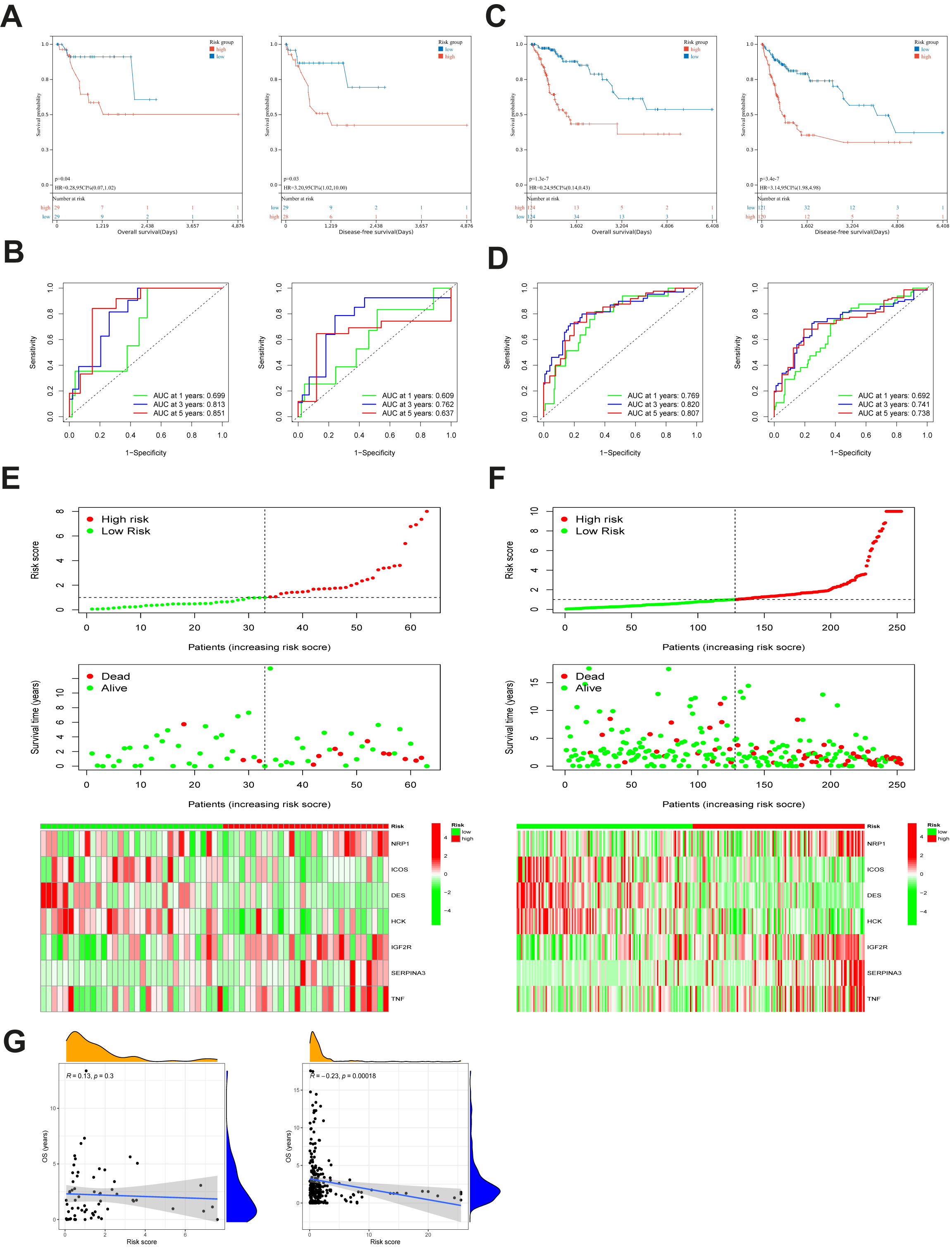

Supplement: Supplementary file 4 [file Image1.tif]
