# Supplementary material for: A panel of seven immune-related genes can serve as a good predictive biomarker for cervical squamous cell carcinoma
Source: Front Genet. 2022 Nov 2;13:1024508. doi: 10.3389/fgene.2022.1024508 (PMC9667556; doi:10.3389/fgene.2022.1024508)
Supplement: Supplementary file 5 [file Image4.pdf]

SUPPLEMENTARY FIGURE 1. Validation of the signature in testing set and the whole TCGA-CSCC set. **(A)** OS curves (left) and DFS curves (right) of high- and low-risk groups stratified by the prognostic signature in testing set. **(B)** ROC curves of the testing set corresponding to 1-, 3-, and 5-year OS (left) and DFS (right). **(C)** OS curves (left) and DFS curves (right) of high- and low-risk groups stratified by the prognostic signature in the whole TCGA-CSCC set. **(D)** ROC curves of the whole TCGA-CSCC set corresponding to 1-, 3-, and 5-year OS (left) and DFS (right). **(E,F)** The risk score distribution, survival status of patients, and expression heatmap of seven signature genes (from top to bottom) in testing set (left) and the whole TCGA-CSCC set (right). **(G)** The correlation between OS and risk scores in testing set (left) and the whole TCGA-CSCC set (right).

SUPPLEMENTARY FIGURE 2. External validation of the signature in GSE44001. **(A)** DFS curves of different risk groups in GSE44001. **(B)** ROC curves of the GSE44001 corresponding to 1-, 3-, and 5-year DFS. **(C)** The correlation between DFS and risk scores in GSE44001. **(D)** The risk score distribution, survival status of patients, and expression heatmap of seven signature genes (from top to bottom) in GSE44001.

SUPPLEMENTARY FIGURE 3. Functional enrichment analysis and survival analysis of different infiltrated immune cells. GO enrichment analysis of prognostic IR-DEGs, including biological processes analysis **(A)**, cellular components analysis **(B)** and molecular functions analysis **(C)**. **(D)** KEGG enrichment analysis of prognostic IR-DEGs. **(E)** Taking the median expression of different infiltrated immune cells as the cutoff value, survival analysis was conducted.
