# Supplementary material for: A panel of seven immune-related genes can serve as a good predictive biomarker for cervical squamous cell carcinoma
Source: Front Genet. 2022 Nov 2;13:1024508. doi: 10.3389/fgene.2022.1024508 (PMC9667556; doi:10.3389/fgene.2022.1024508)
Supplement: Supplementary file 6 [file Table2.docx]

**Supplementary Table2. Univariate Cox analysis and Multivariate Cox analysis in the whole TCGA-CSCC dataset**

|  | Univariate Cox analysis | | | |  | Multivariate Cox analysis | | | |
| --- | --- | --- | --- | --- | --- | --- | --- | --- | --- |
| id | HR | HR.95L | HR.95H | pvalue |  | HR | HR.95L | HR.95H | pvalue |
| Age | 1.001732 | 0.968214 | 1.03641 | 0.920635 |  | 0.991337 | 0.955011 | 1.029043 | 0.647792 |
| Grade | 0.746259 | 0.385134 | 1.445996 | 0.385824 |  | 0.693962 | 0.330837 | 1.45565 | 0.333743 |
| T | 1.635053 | 0.904154 | 2.956795 | 0.103816 |  | 1.537844 | 0.745859 | 3.170795 | 0.243718 |
| N | 2.845247 | 1.286381 | 6.293181 | 0.00983 |  | 3.268476 | 1.439178 | 7.422942 | 0.004656 |
| riskScore | 1.140068 | 1.072406 | 1.212 | 2.68E-05 |  | 1.166256 | 1.09238 | 1.245129 | 4.10E-06 |
